# Cluster expansion of *apolipoprotein D* (*ApoD*) genes in teleost fishes

**DOI:** 10.1186/s12862-018-1323-x

**Published:** 2019-01-08

**Authors:** Langyu Gu, Canwei Xia

**Affiliations:** 1 Key Laboratory of Freshwater Fish Reproduction and Development (Ministry of Education), Key Laboratory of Aquatic Science of Chongqing, School of Life Sciences, Southwest University, Chongqing, China; 2 Zoological Institute, Department of Environmental Sciences, University of Basel, Basel, Switzerland; 3 Ministry of Education Key Laboratory for Biodiversity and Ecological Engineering, College of Life Sciences, Beijing Normal University, Beijing, China

**Keywords:** *Apolipoprotein D* (*ApoD*), Duplication, Gene cluster, Positive selection, Breakpoints, Teleost fishes

## Abstract

**Background:**

Gene and genome duplication play important roles in the evolution of gene function. Compared to individual duplicated genes, gene clusters attract particular attention considering their frequent associations with innovation and adaptation. Here, we report for the first time the expansion of the *apolipoprotein D* (*ApoD*) ligand-transporter genes in a cluster manner specific to teleost fishes.

**Results:**

Based on comparative genomic and transcriptomic analyses, protein 3D structure comparison, positive selection detection and breakpoints detection, the single *ApoD* gene in the ancestor expanded into two clusters following a dynamic evolutionary pattern in teleost fishes. Orthologous genes show conserved expression patterns, whereas lineage-specific duplicated genes show tissue-specific expression patterns and even evolve new gene expression profiles. Positive selection occurred in branches before and after gene duplication, especially for lineage-specific duplicated genes. Cluster analyses based on protein 3D structure comparisons, especially comparisons of the four loops at the opening side, show gene duplication-segregating patterns. Duplicated *ApoD* genes are predicted to be associated with forkhead transcription factors and *MAPK* genes. *ApoD* clusters are located next to the breakpoints of genome rearrangements.

**Conclusions:**

Here, we report the expansion of *ApoD* genes specific to teleost fishes in a cluster manner for the first time. Neofunctionalization and subfunctionalization were observed at both the protein and expression levels after duplication. Evidence from different aspects—i.e., abnormal expression-induced disease in humans, fish-specific expansion, predicted associations with forkhead transcription factors and *MAPK* genes, specific expression patterns in tissues related to sexual selection and adaptation, duplicated genes under positive selection and their location next to the breakpoints of genome rearrangements—suggests the potentially advantageous roles of *ApoD* genes in teleost fishes. The cluster expansion of *ApoD* genes specific to teleost fishes provides thus an ideal evo-devo model for studying gene duplication, cluster maintenance and new gene function emergence.

## Background

Gene and genome duplication play important roles in evolution by providing new genetic materials [1]. The gene copies emerging from duplication events (including whole genome duplications (WGD)) can undergo different evolutionary fates, and a number of models have been proposed as to what can happen after duplication [2]. In many instances, one of the duplicates becomes silenced via the accumulation of deleterious mutations (i.e. pseudogenization or nonfunctionalization [1]). Alternatively, the original pre-duplication function can be subdivided between duplicates (i.e. subfunctionalization) [3], or one of the duplicates can gain a new function (i.e. neofunctionalization) [4]. Although the probability of accumulating beneficial substitutions is relatively low, there are, examples of neofunctionalization. For example, the duplication of *dachshund* in spiders and allies has been associated with the evolution of a novel leg segment [5]; the expansion of repetitive regions in a duplicated trypsinogen-like gene led to functional antifreeze glycoproteins in Antarctic notothenioid fish [6]; and the duplication of *opsin* genes is implicated in trichromatic vision in primates [7]. Another selective advantage of gene duplication can be attributed to increased numbers of gene copies, e.g. in the form of gene dosage effects [8, 9]. Multiple mechanisms can act together to shape different phases of gene evolution after duplication [10].

Functional changes after gene duplication can occur at the protein level [6, 11, 12]. For example, the physiological division of labour between the oxygen-carrier function of haemoglobin and the oxygen-storage function of myoglobin in vertebrates (subfunctionalization) [13] and the acquired enhanced digestive efficiencies of the duplicated gene encoding of pancreatic ribonuclease in leaf monkeys (neofunctionalization) [14]. However, the probability that functional mutations can occur in a coding region is relatively small due to pleiotropic constraints. Instead, changes at the expression level are more tolerable and can offer immediate phenotypic consequences [15, 16]. For example, complementary degenerative mutation in the regulatory regions of duplicated genes is a common mechanism for subfunctionalization [2, 17]. Many examples have provided evidence that duplicated genes acquiring new expression domains are linked to neofunctionalization (e.g., *dac2*, a novel leg segment in arachnids [5]; *elnb*, bulbus arteriosus in teleost fishes [18]; and *fhl2b*, egg-spots in cichlid fishes [19]).

In some cases, gene clusters resulting from gene duplication have attracted considerable attention, such as Hox gene clusters [20], globin gene clusters [21], paraHox gene clusters [22], MHC gene clusters [23] and opsin gene clusters [24]. Duplicated genes in clusters are usually related to innovation and adaptation [11, 24, 25], suggesting advantageous roles during evolution. The expansion of gene clusters can be traced back to WGD and tandem duplication [25, 26]. In addition to the two rounds of WGD that occurred before the split between cartilaginous and bony fish [27], the ancestor of teleost fishes experienced another round of WGD (teleost genome duplication, TGD) after divergence from non-teleost actinopterygians, including bichir, sturgeon, bowfin and spotted gar [28, 29]. This extra TGD provides an additional opportunity for gene family expansion in fishes [24, 30–32].

Genome rearrangements have been suggested to occur frequently in teleost fishes [33]. If genome rearrangements can capture locally adapted genes or antagonist sex-determining genes by reducing recombination, the rearranged genome can promote divergence and reproductive isolation [34, 35] and thus contribute to speciation and adaptation. Examples can be found in butterflies [36], mosquitoes [37] and fish [38]. This occurs especially when advantageous genes are located next to the breakpoints of a genome rearrangement due to the associated low recombination rates [39, 40]. Considering that the expansion of gene clusters is usually adaptive (as mentioned above) and is linked to genome instability [35, 41, 42], it will be interesting to investigate the roles of gene clusters located next to the breakpoints of genome rearrangements. However, related studies are sparse.

Here, we report for the first time the cluster expansion of *apolipoprotein D* (*ApoD*) in teleost fishes. The *ApoD* gene belongs to the lipocalin superfamily of lipid transport proteins [43, 44]. In humans, ApoD is suggested to function as a multi-ligand, multifunctional transporter (e.g., hormone and pheromone transporter) [44, 45], which is important in homeostasis and in the housekeeping of many organs [45]. It is expressed in multiple tissues, most notably in the brain and testis (see e.g. [44, 46, 47]) and is involved in the central and peripheral nervous systems [44]. However, no detailed analyses of the *ApoD* gene in fishes have been reported yet. Here, we investigate the evolutionary history of *ApoD* genes in fishes for the first time.

## Results

### *In silico* screening and phylogenetic reconstruction of *ApoD* genes

**Fig. 1:**
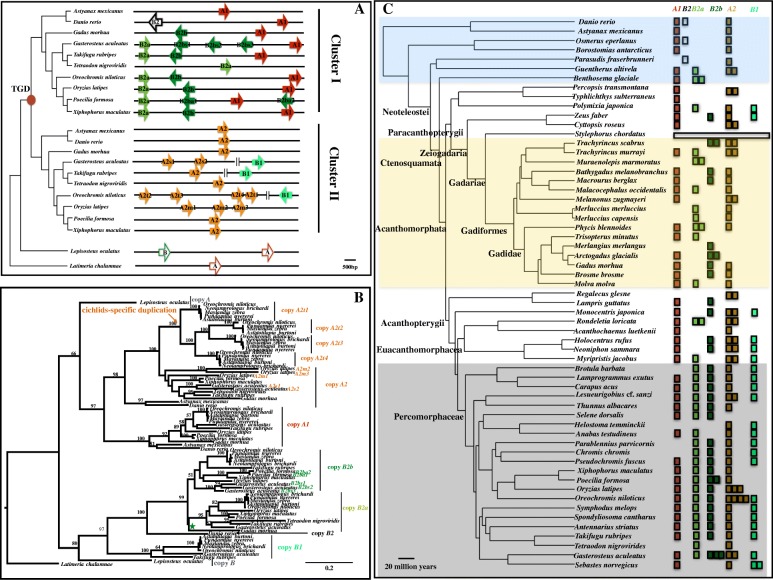
**a** Cluster expansion of *ApoD* genes specific to teleost fishes after teleost genome duplication (TGD). Each arrow represents a single gene copy. Genes highlighted in red and orange represent paralogs derived from one common ancestor. Genes colored dark and light green represent paralogs derived from one common ancestor. Phylogeny reconstruction is based on a consensus fish phylogeny [24]. **b** Maximum likelihood phylogenetic tree reconstruction to infer gene duplication. Bootstrap values > 50% are marked on the branch. Lineage-specific duplication events are labelled. Note that although copy *B2b* of *Gadus morhua* is clustered within the *B2a* clade (labelled with a green star), its bootstrap value is low, which could be due to recent duplication. **c** The dynamic evolutionary pattern of *ApoD* genes across the phylogeny of teleost fishes. Highly variable copy numbers are detected in different lineages, especially in the Paracanthopterygii lineage. *Stylephorus chordatus* has lost all *ApoD* genes. Compared to copy *A1*, copy *A2* exhibits more variable lineage-specific duplicates in different fishes, with the highest numbers appearing in tilapia (four copies). Copy *B1* is absent in the whole clade of Gadiformes. Copy *B2* only shows up in the species *Danio rerio*, *Osmerus eperlanus* and *Parasudis fraserbrunneri*. The co-existence of *B2a* and *B2b* is common in Percomorphaceae. The largest numbers of lineage-specific duplicated genes are found in tilapia (copy *A2*), medaka (copy *A2*) and stickleback (copy *B2b*) in Percomorphaceae

To investigate the expansion of *ApoD* genes, we performed phylogenetic reconstruction with high-quality assembly of genomes (Fig. 1a, b). There is one *ApoD* gene in coelacanth (*Latimeria chalumnae*), and two copies (*A* and *B*) in spotted gar (*Lepisosteus oculatus*) located in one cluster. Different numbers of *ApoD* genes are located in two clusters in different teleost fishes, i.e., two copies in cavefish (*Astyanax mexicanus*; *A1* and *A2*) and in tetraodon (*Tetraodon nigroviridis*; *B2a* and *A2*); three copies in zebrafish (*Danio rerio*; *A1, A2* and *B2*) and in cod (*Gadus morhua*; *A1, A2* and *B2b*); four copies in platyfish (*Xiphophorus maculatus*; *A1*, *A2*, *B2a* and *B2b*); five copies in Amazon molly (*Poecilia formosa*; *A1*, *A2*, *B2a*, *B2ba1*, *B2ba2*) and in fugu (*Takifugu rubripes*; *A1*, *A2*, *B1*, *B2a*, *B2b*); six copies in medaka (*Oryzias latipes*; *A1*, *A2m1*, *A2m2*, *A2m3*, *B2a*, *B2b*); and eight copies in stickleback (*Gasterosteus aculeatus*; *A1*, *A2s1*, *A2s2*, *B1*, *B2a*, *B2bs1*, *B2bs2*, *B2bs3*) and in tilapia (*Oreochromis niloticus*; *A1*, *A2t1*, *A2t2*, *A2t3*, *A2t4*, *B1*, *B2a*, *B2b*). Noticeably, although the copy *B2b* in cod is clustered within the *B2a* clade based on a maximum likelihood (ML) tree, the bootstrap value is very low, which could be due to recent duplication (Fig. 1b). Considering its gene direction and syntenic nature relative to *B2b* genes in other species (Fig. 1a), we named it copy *B2b*. Sequence alignment for the tree construction can be found in 10.5061/dryad.39g63v2 [116].

To further retrieve the evolutionary history of *ApoD* genes in fishes, we performed an *in silico* screen across the whole phylogeny of teleost fishes using draft genomes (Fig. 1c). No *ApoD* gene was detected in *Stylephorus chordatus*. Unlike copy *A1*, which shows no lineage-specific duplication, copy *A2* exhibits variable lineage-specific copies in different fishes, with the highest number appearing in a cichlid fish, tilapia (four copies). Copy *B1* is absent in the clade of Gadiformes but is kept in Acanthopterygii. Species in *Danio rerio*, *Osmerus eperlanus* and *Parasudis fraserbrunneri* possess copy *B2*. The co-existence of *B2a* and *B2b* is common in Percomorphaceae. The largest numbers of lineage-specific duplicated *ApoD* genes were found in tilapia (copy *A2*), medaka (copy *A2*) and stickleback (copy *B2b*) in Percomorphaceae. The predicted *ApoD* gene sequences can be found in 10.5061/dryad.39g63v2 [116].

**Fig. 2:**
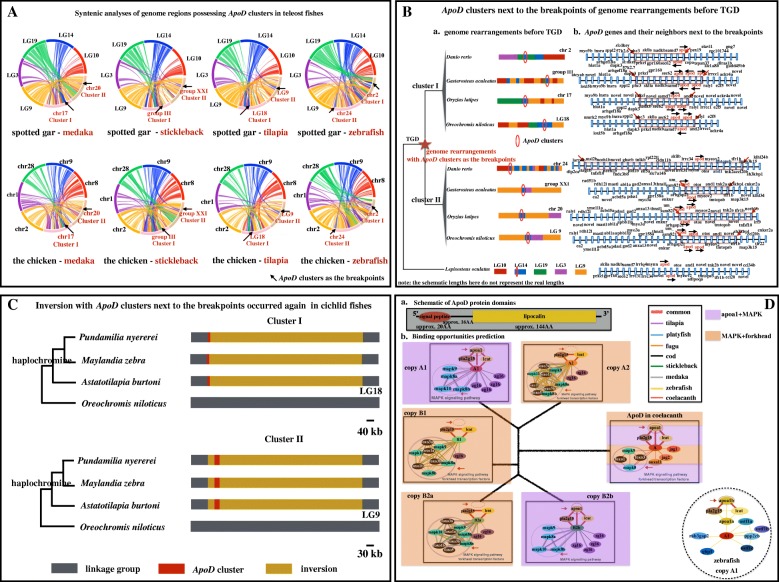
(**a**) Syntenic analyses of genome regions possessing *ApoD* clusters. The same color between spotted gar and the chicken represents orthologous chromosomes. Two paralogous duplicated segments in teleost fishes (chromosomes (chrs)/linkage groups (LGs) indicated in red) can be traced back to one corresponding orthologous region in spotted gar and the chicken (chrs/LGs labelled in black), linked by colorful lines. Arrows show the regions in which *apolipoprotein D* (*ApoD*) clusters are located in. (**b**) *ApoD* clusters next to the breakpoints of genome rearrangements before teleost genome duplication (TGD). The same color between spotted gar and the chicken represents orthologous chromosomes. The red arrows show breakpoints, and the black arrows show gene directions. Neighboring genes are named according to the Ensembl database. (**c**) Inversion with *ApoD* clusters next to the breakpoints occurred again in cichlid fishes. The haplochromine lineage, the most species-rich lineage of cichlid fishes, is labelled. (**d**) ApoD domains and protein-protein association predictions. a. Conserved domains include a single peptide of approx. 20 amino acids (AA) and a lipocalin domain of approx. 144 AA. b. Different paralogs exhibited differential associations. The common associations are with *pla2g15*, *lcat* and *MAPK* genes. One class (copies *A2*, *B2a* and *B1*) is associated with multiple forkhead transcription factors. The other class (copies *A1* and *B2b*) lost this association. Instead, it is associated with the lipoprotein-related gene *apoa1*. The single *ApoD* gene in coelacanth possesses both associations

To infer the relationship between *ApoD* gene duplication and TGD, syntenic analyses were conducted. These revealed that two paralogous regions in teleost fishes correspond to one ohnologous region in spotted gar and the chicken (*Gallus gallus*) (connected by the same colored lines in Fig. 2A). For example, the regions highlighted in yellow in linkage group (LG) 9 in spotted gar (left above), or on chicken chromosome (chr) 2 (bottom left) have paired paralogous regions in medaka. These are found on chr17, where the *ApoD* cluster I is located, and on chr20, where the *ApoD* cluster II is located. Similarly, the region highlighted in purple in LG3 of spotted gar and on chicken chr1 has paired paralogous regions (connected by purple lines) on chr17 and chr20 in medaka.

### *ApoD* clusters next to the breakpoints of genome rearrangements

Syntenic analyses clearly show that more than one chromosome in spotted gar and the chicken are syntenic to the corresponding paralogous regions in which *ApoD* clusters are located in teleost fishes. For example, chromosomes (or LGs) containing *ApoD* clusters (chr17 and chr 20 in medaka, LG III and LG XXI in stickleback, LG9 and LG18 in tilapia, and chr2 and chr24 in zebrafish) are syntenic to chromosomes LG10, LG9, LG19, LG3 and LG14 in spotted gar and chr8, chr2, chr28, chr1 and chr9 in the chicken (Fig. 2A and B). Noticeably, *ApoD* clusters are located next to the breakpoints of genome rearrangements (Fig. 2A and B). The rearranged segments (approx. 80 kb to 200 kb syntenic with LG14 in spotted gar, Fig. 2B) include *ApoD* clusters and their neighboring genes (e.g., *and2*, *samd7*, *sec62*, *nadkb*, *gpr160*, *skila*, *prkci* and *phc3* for Cluster I; *otos*, *myeov2*, *and1*, *tmtopsb*, *tnk2a* and *tfr1b* for Cluster II) (Fig. 2B). Analyses of available cichlid genomes further show that inversion of the segments (approx. 600 kb to 800 kb) with *ApoD* clusters as the breakpoints also occurred in species belonging to the most species-rich lineage of cichlid fishes, the haplochromine lineage, as supported by split-read analyses using Delly (Fig. 2C).

### Protein-protein association predictions and protein 3D structure comparisons

To assess the biological functions of different *ApoD* genes, protein-protein associations were predicted. Protein domain architecture analyses revealed that ApoD proteins in different fishes are composed of a signal peptide of approx. 20 amino acids and a lipocalin domain of approx. 144 amino acids (Fig. 2D). The common associations of *ApoD* genes are with the immunity-related gene *pla2g15* [48, 49], the high-density lipoprotein biogenesis-related gene *lcat* [50], different copies of *zg16* related to pathogenic fungi recognition [51] and *MAPK* genes [52, 53]. *ApoD* genes in teleost fishes can be subdivided into two classes based on the association predictions. *ApoD* genes from one class (copy *A2*, copy *B2a* and copy *B1*) are associated with forkhead transcription factors. *ApoD* genes from the other class (copy *A1* and copy *B2b*) lost their associations with forkhead transcription factors but are associated with the lipoprotein-related gene *apoa1* [54]. Noticeably, the only *ApoD* gene in coelacanth possesses associations with both forkhead transcription factors and *apoa1*. *ApoD* in coelacanth is also associated with genes encoding ligands that can activate the Notch signalling pathway (*jag1*, *jag2*) [55], and the gene belongs to the annexin family (*anxaII*) (Fig. 2D). Noticeably, more members of forkhead transcription factors and *MAPK* genes are associated with *ApoD* genes after duplication. Unlike in other fishes, copy *A1* in zebrafish is associated with bone resorption-related duplicated genes (*ostf1a*, *ostf1b*, *ostf1c*) [56], the cell growth and division-related gene *ppp2cb* [57], the neurodevelopment-related gene *rab3gap2* [58] and the guanylate-binding gene *gbp1* [59] (Fig. 2D).

**Fig. 3:**
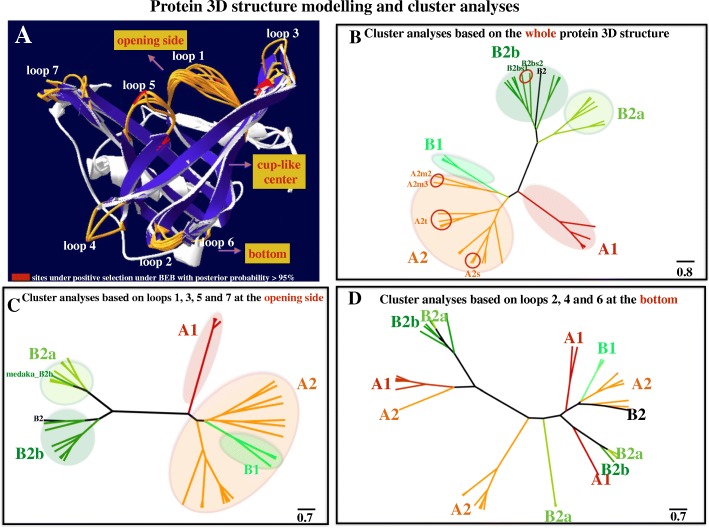
**a** Protein 3D structure modelling. Different ApoDs among species exhibit a conserved 3D structure, including a cup-like central part made up of eight antiparallel *β*-sheet strands and two ends connected by loops (a wide opening part formed by loops 1, 3, 5 and 7 and a narrow closed bottom formed by loops 2, 4 and 6). Unlike the very conserved cup-like central part, the loops at the two ends are highly variable. Note that most sites under positive selection are located on the loops or on the connections between the loops and the cup-like central part. **b** Cluster analyses based on the whole protein 3D structure. Cluster analyses can clearly segregate different *ApoD* duplicates, including lineage-specific duplicated genes. **c** Cluster analyses based on loops 1, 3, 5 and 7 at the opening side. Cluster analyses can segregate different *ApoD* duplicates. **d** Cluster analyses based on loops 2, 4 and 6 at the bottom. This cluster analyses cannot segregate different duplicates

Homology protein structure modelling of different *ApoD* genes shows a conserved structure, including a cup-like central part made up of eight antiparallel *β*-sheet strands and two ends connected by loops (a wide opening side formed by loops 1, 3, 5 and 7 and a narrow closed bottom formed by loops 2, 4 and 6; Fig. 3a). Interestingly, unlike the very conserved cup-like central part, the loops are highly variable (Fig. 3a). Cluster analyses based on the whole protein 3D structure can clearly segregate different duplicates. This is especially true of lineage-specific duplicated genes that are clustered together. Copy *B1s* is clustered nested within the copy *A2* clade (Fig. 3b). The same analyses focused on only the four loops (loops 1, 3, 5 and 7) at the opening side show a similar segregation pattern (Fig. 3c). However, cluster analyses focused only on the three loops (loops 2, 4 and 6) at the bottom side did not show a gene duplication-segregation pattern (Fig. 3d). Details concerning the PDB files and the cluster results can be found in 10.5061/dryad.39g63v2 [116].

### Positive selection detection of *ApoD* genes

**Fig. 4:**
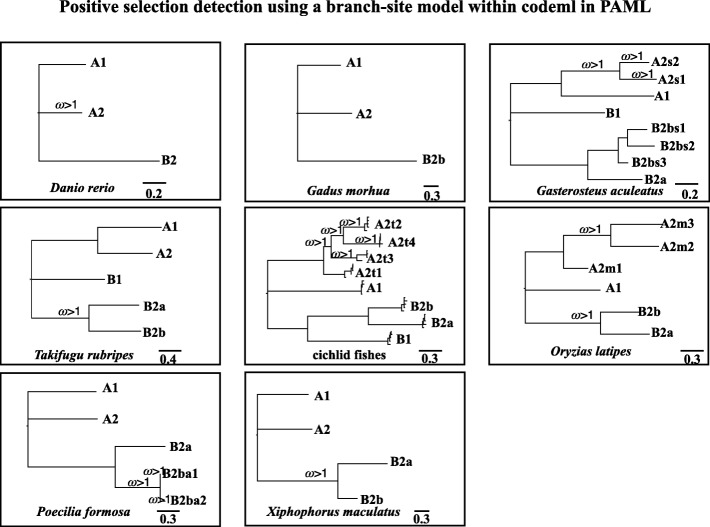
Positive selection detection using a branch-site model within codeml in PAML. Many duplicated *ApoD* genes are under positive selection, with a value of ω significantly larger than 1 after Bonferroni correction, especially for lineage-specific duplicated genes. Note that if the whole clade is designated as the foreground branch when detecting positive selection, each branch of this clade will be labelled if its ω value is significantly larger than 1. The unrooted tree was used for PAML analyses, and the rooted tree here is only for presentation. More details can be found in 10.5061/dryad.39g63v2 [116]

**Fig. 5:**
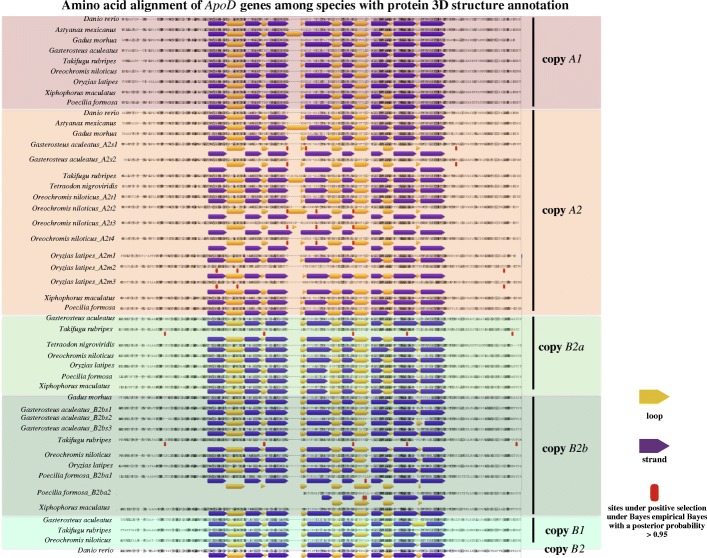
Amino acid alignment of *ApoD* genes among species with protein 3D structure annotation. The purple arrow represents the *β*-strand, and the yellow arrow represents the loop. Sites under positive selection under Bayes empirical Bayes (BEB) with a posterior probability > 95% are labelled with a red rectangle. Most sites under positive selection are distributed on the loops or on the connections between the loop and the *β*-strand. Note that the positions of the sites under positive selection are based on the codon alignment used for PAML analyses (10.5061/dryad.39g63v2 [116]) instead of on the amino acid alignment here, but the amino acids that under positive selection are the same in Fig. 5 and 10.5061/dryad.39g63v2 [116]

Positive selection detection using a branch-site model within codeml in PAML revealed that positive selection occurred in branches before and after duplication, for example, the branch of copy *A2* in zebrafish; the ancestral branch of copies *B2a* and *B2b* in fugu, medaka and platyfish; and, especially, in lineage-specific duplicated genes, such as in stickleback, cichlid fishes and Amazon molly (Fig. 4 and 10.5061/dryad.39g63v2 [116]). Positive selection sites under Bayes empirical Bayes (BEB) with a posterior probability > 95% are shown in Fig. 5 and 10.5061/dryad.39g63v2 [116]. Noticeably, most sites under positive selection are located on the highly variable loops or on the connections between loops and the cup-like central part at the opening side (loops 3 and 5; Figs. 3a and 5). Multiple tests of positive selection on all possible foreground branches followed by Bonferroni correction is a relatively conservative strategy. If we only focus on one prior hypothesis on a particular branch without doing multiple test corrections, we will find more branches with dN/dS values significantly larger than 1 (*p* < 0.05; 10.5061/dryad.39g63v2 [116]). However, we chose to use our strategy to make the analyses more rigorous. Even with this strict but comprehensive strategy, our results still show that positive selection occurred in branches before and after gene duplication, especially for lineage-specific duplicated genes. Details about the codon alignment for the PAML analyses can be found in 10.5061/dryad.39g63v2 [116].

### Gene expression profile detection of *ApoD* genes in different species

**Fig. 6:**
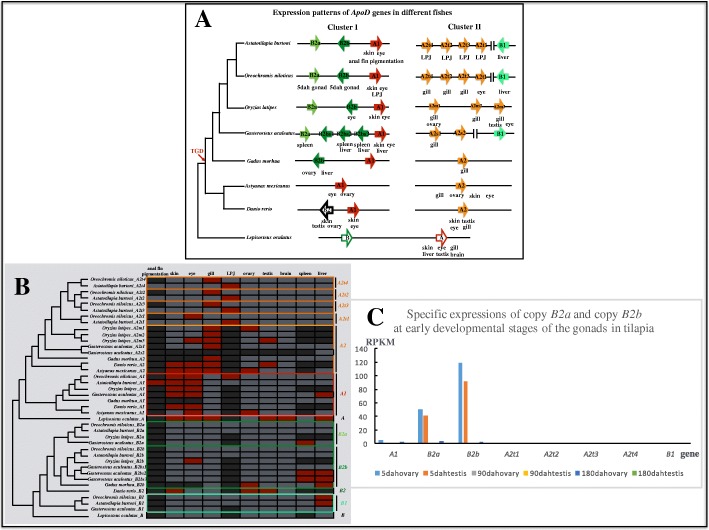
Gene expression profiles of different *ApoD* genes. **a** Expression patterns of *ApoD* genes in different fishes. Each block represents a single gene copy. The phylogeny reconstruction was based on the consensus fish phylogeny from a previous study [24]. **b** Detailed gene expression profiles of *ApoD* genes among fishes. Red, tissues with a high expression level; dark grey, data unavailable (either because the tissue does not exist in the species or is undetected in this study); light grey, tissues with a low expression level. LPJ, the lower pharyngeal jaw in cichlid fishes. TGD, teleost genome duplication. **c** Specific expressions of copy *B2a* and copy *B2b* at early developmental stages of the gonads in tilapia. Analyses are based on the available transcriptomic data from a previous study [117]. RPKM, reads per kilobase per million mapped reads; dah, days after hatching

Based on the available raw transcriptomic data analyses and the results from quantitative polymerase chain reaction (qPCR), the gene expression profiles of *ApoD* genes were detected in different species (Fig. 6). Copy *A1* is mainly expressed in the skin and the eye. Noticeably, copy *A1* is also highly expressed in the novel anal fin pigmentation patterns of the cichlid fish, *Astatotilapia burtoni*, which is consistent with the findings of our previous study [60]. Copy *A2* shows redundant expression patterns in the gills, eye, skin and gonads in zebrafish and cavefish, but its orthologous *A2* genes, including lineage-specific duplicated genes in the species of Acanthomorphata tested herein (cod, stickleback, medaka, tilapia and *A. burtoni*) are expressed in the gills and in the related apparatus, the lower pharyngeal jaw. Copy *B2* in zebrafish shows redundant expression profiles (in the skin and gonads) overlapping with the profiles of copies *A1* and *A2*. Copies *B2a* and *B2b* show specific expression patterns in different fishes of Acanthomorphata tested herein. For example, copy *B2b* is expressed in the ovary and in the liver in cod, whereas *B2a* and *B2bs* are mainly expressed in the spleen and in the liver of the stickleback. The expression of *B2a* and *B2b* was not detected in adult medaka, tilapia and *A. burtoni*. Interestingly, based on the available transcriptomic data for the different developmental stages of the gonads in tilapia, we found that *B2a* and *B2b* are highly specifically expressed at the early developmental stage of gonad tissues (5 days after hatching, 5dah; Fig. 6). Copy *B1* is highly specifically expressed in the liver in tilapia and in *A. burtoni* but not in stickleback in which *B1* is inverted (Fig. 1a). Noticeably, no expression profile was detected for copy *B* in spotted gar, at least, not in the tissues we tested. Instead, expression in tissues including the skin, eye, gill, liver, testis and brain were detected for copy *A* in spotted gar. Details can be found in Fig. 6 and 10.5061/dryad.39g63v2 [116].

## Discussion

### Dynamic evolution of *ApoD* genes in teleost fishes

Here, we report the cluster expansion of *ApoD* genes specific to teleost fishes for the first time. Phylogenetic reconstruction and syntenic analyses clearly show the expansion of *ApoD* genes into two clusters with lineage-specific tandem duplications after TGD. The interplay between genome duplication and tandem duplication to prompt the expansion of the gene family has already been shown in a few studies [61–63]. It has been suggested that the fixation of duplications is much more common in genome regions in which the rates of mutations are elevated due to the presence of already-fixed duplication, which is the so-called “snow-ball” effect [64]. Both genome duplication and tandem duplication can produce raw genetic materials to fuel the diversification of teleost fishes at later stages, e.g., morphological complexity and ecological niche expansion, which is compatible with the “*time lag model*” [65]. Neofunctionalization and subfunctionalization after duplication are important steps to realize this diversification, and both can be detected in *ApoD* duplicates.

The functional divergence of *ApoD* genes occurred at the protein level. On one hand, based on the protein-protein association predictions, although common associations with *MAPK* genes are shared among *ApoD* genes, subdivided associations with forkhead transcription factors and *apoa1* were detected in different paralogous *ApoD* genes, indicating subfunctionalization. Noticeably, more members of forkhead transcription factors and *MAPK* genes are associated with *ApoD* genes after duplication. In this case, neofunctionalization could also occur but was not limited to the dosage effects. On the other hand, divergence was also detected at the protein-structure level. Protein structures are often related to functional divergence [66–69]. New folds can even evolve novel functions [70, 71]. Even mutations of a few residues can induce structural changes [72, 73]. Therefore, structure-based inference is important for understanding molecular function. Our protein 3D structure modelling shows a conserved backbone conformation, in spite of sequence divergence. Indeed, this is a feature of the lipocalin family [67], to which ApoDs belong [43, 44, 74]. The cup-like central part is used to transport large varieties of ligands, such as hormones and pheromones [74]. Interestingly, cluster analyses based on the whole protein structure or on only the four loops at the opening side can segregate different duplicates, although not for copy *B1.* Considering that copy *B1* has been lost multiple times, its function might not be essential and could share functions with other copies; thus, it is not surprising if it is clustered together within the copy *A2* clade. The cluster results of two other individual genes (copy *B2* of zebrafish and *B2b* of medaka) do not affect the general segregation pattern. However, unlike the four loops at the opening side, cluster analyses based on only the three loops (loops 2, 4 and 6) at the bottom side did not show a clear segregation pattern. It has been suggested that the loops of lipocalin proteins can affect ligand binding specificities, which are similar to the binding modes of antibodies [75], and mediate protein-protein interactions [67]. Their segregation with different duplicates might indicate that functional divergence occurred at the loops at the opening side. The evidence that most amino acids under positive selection are located on the loops or connections between loops and strands further indicates the potentially important roles of these loops during evolution. Actually, reshaping different parts of the protein structure is an efficient way to produce functional divergence at the protein level within a short time [74], and this could be one of the ways by which divergence at the protein level occurred in ApoDs.

The functional divergence of *ApoD* genes also occurred at the expression level. Different *ApoD* genes in zebrafish and cavefish exhibited redundant expression profiles but became more specific as the numbers of tandem duplicates increased, indicating subfunctionalization. Noticeably, new expression profiles were detected in duplicated paralogs, e.g., copy *A1* in novel anal fin pigmentation patterns and copy *A2s* in the lower pharyngeal jaw in *A. burtoni*, which belongs to the most species-rich cichlid fish lineage, the haplochromine lineage. These two traits are key innovations associated with adaptive radiation in cichlid fishes [76]. With expression changes, duplication can be an important source for the emergence of novelty [77], especially if they are adaptive. The relaxed selection pressure induced by the changing expression profiles can even further prompt the accumulation of mutations at the protein level.

### Potential advantageous roles of *ApoD* genes in teleost fishes

The *ApoD* gene has been thoroughly studied in human and mice, and its abnormal expression was reported to be related to human diseases, such as Parkinson’s disease and Alzheimer’s disease [78–80]. Interestingly, as our study revealed here, *ApoD* dynamically expanded in fishes into two clusters on different chromosomes. Furthermore, these expanded duplicates are transcriptionally active. For example, they are highly expressed and are restricted to different tissues related to sexual selection (skin [81], eye [82, 83], gonads, and anal fin pigmentation patterns [60]) and adaptation (gills [84–87], spleen [88, 89], and lower pharyngeal jaw [76, 90]). These tissues are also derived from the neural crest, which is a key innovation in vertebrates [76, 91–93]. Additionally, many duplicated *ApoD* genes are under positive selection, especially for lineage-specific duplicated genes. Combined with their associations with *MAPK* genes and forkhead transcription factors and their functions as pheromone and hormone transporters, we report evidence that suggests the potential importance of *ApoD* genes in fishes.

Noticeably, two clusters exhibited distinct expression patterns. *ApoD* genes in cluster I in most fishes are expressed in tissues related to sexual selection (skin, eye, gonad and anal fin pigmentation), but in cluster II, *ApoD* genes are mainly expressed in tissues related to adaptation (gills and lower pharyngeal jaw). Interestingly, both clusters were maintained with their neighboring genes and are located next to the breakpoints of genome rearrangements during evolution. An inversion of a chromosomal section containing *ApoD* clusters next to breakpoints occurred again in the haplochromine lineage, the most species-rich lineage of cichlid fishes [94]. If genome rearrangements can capture locally adaptive genes or genes related to sexual antagonism, it could accelerate divergence by reducing recombination rates, thus prompting speciation and adaptation and even the emergence of neo-sex chromosomes [95]. Given that many *ApoD* genes are under positive selection, their location next to the breakpoints of genome rearrangements gives them more opportunities to be involved in speciation and adaptation, which deserves further investigation.

## Conclusions

Here, we report the cluster expansion of *ApoD* genes in a cluster manner specific to teleost fishes for the first time. Different types of evidence based on computational evolutionary analyses strongly suggest the potentially advantageous roles of *ApoD* genes in fishes. An in-depth functional characterization of *ApoD* genes could help consolidate a model for the study of subfunctionalization and neofunctionalization. Moreover, finding the regulatory mechanisms behind the cluster expansion of *ApoD* genes and the reason why the *ApoD* gene expansion was specific to fishes remain open questions. As more fish genomes with high assembly quality become available, especially those of closely related species such as cichlid fishes, the roles of *ApoD* genes in fish speciation and adaptation can be further investigated. Above all, the *ApoD* clusters reported here provide an ideal evo-devo model for studying gene duplication, cluster maintenance, and the gene regulatory mechanism and their roles in speciation and adaptation.

## Methods

### *In silico* screening and phylogenetic reconstruction to infer gene duplication

To retrieve *ApoD* gene duplication in teleost fishes, we first extracted orthologs and paralogs in fishes with available genome data from Ensembl (Release 84) [96] and the NCBI database (https://www.ncbi.nlm.nih.gov/genome/). To confirm gene copy numbers, all orthologs and paralogs were used as queries in a tblastx search against the corresponding genomes. For all unannotated positive hits, a region spanning approx. 2 kb was extracted, and open reading frames (ORF) were predicted using Augustus (http://augustus.gobics.de/) [97]. A BLAST search was then performed to compare the predicted coding sequences with the existing transcriptome database to retrieve the corresponding cDNA sequences. The cDNAs were then re-mapped to the corresponding predicted genes to re-check the predicted exon-intron boundaries. Coding sequences of genes from humans and spotted gar, as well as those of neighboring genes, were used to perform a tblastx search against the genomes of lamprey (*Lampetra fluviatilis*) and amphioxus (*Branchiostoma belcheri*). To infer gene duplication, an ML analysis was performed using *ApoD* genes retrieved from available assembled genomes in RAxML v8.2.10 [98] with the GTR + G model and the ‘-f a’ option, which generates the optimal tree and conducts 10,000 rapid bootstrap searches.

To further retrieve *ApoD*s from the draft genomes of other fishes across the whole phylogeny [23], all sequences retrieved above were used as queries in a tblastx search using a threshold *e* value of 0.001. The hit scaffolds were retrieved, and genes within the scaffolds were predicted using Augustus. The predicted *ApoD*s were then translated and re-aligned with known *ApoD* ORFs to further confirm the exon-intron boundaries. All predicted orthologs and paralogs were used as a query again in a tblastx search against the corresponding genome data until no more *ApoD* genes were predicted.

### Syntenic analyses and inversion detection

To further confirm the relationship between *ApoD* gene duplication and TGD, gene regions adjacent to the duplicates and to the outgroup species that did not experience TGD (including spotted gar and the chicken) were retrieved. To this end, a window of 5 Mb around the *ApoD* clusters in teleost fishes, as well as the corresponding chromosomes in spotted gar and the chicken, were retrieved from the Ensemble and NCBI databases. Syntenic analyses were performed with SyMap [99]. To further detect the structural variation around the *ApoD* genes in cichlid fishes, we retrieved the available cichlid genome raw data from [100]. The programme Delly [101], based on paired-end split-reads analyses, was used to detect the inversion and its corresponding breakpoints of the segments that contain the *ApoD* clusters in cichlid fishes, with tilapia as the reference.

### Protein-protein interaction prediction, protein 3D structure modelling and comparisons

To predict the biological functions of *ApoD* genes, the protein domains of *ApoD* genes and protein-protein interactions were predicted with the Simple Modular Architecture Research Tool (SMART) [102, 103] and the stringdb database http://string-db.org/ [104]. To determine whether there were divergences at the protein level for different ApoDs, protein 3D structures were simulated with Swiss-model [105–107] using the human ApoD crystal protein structure (PDB ID 2hzr) as the template. The results were further visualized, evaluated and analyzed with Swiss-PdbViewer [108]. To compare the protein structures, we first extracted and converted the corresponding information in the PDB file using Swiss-PdbViewer. Protein 3D structure comparisons were then conducted using Vorolign [109], which can compare closely related protein structures even when structurally flexible regions exist [109]. The protein 3D structure comparisons and cluster analyses were conducted using the ProCKSI-server (http://www.procksi.net/). Clustering results were further visualized using Figtree v1.4.2 (http://tree.bio.ed.ac.uk/software/figtree). Copy *B2ba2* of Amazon molly was not included in the cluster analyses due to its relatively short sequence. The PDB files were used as input files. For the global protein structure comparisons, PDB files were extracted directly from the simulation results of Swiss-model [105–107]. To only obtain PDB files of the loop region, we revised the PDB files manually to get rid of the cup-like central part. To consider the highly variable connections between the loops and the cup-like central part (Fig. 3a), we also included the structures of two more residues next to the loops. The resulting PDB files were further checked using Swiss-PdbViewer.

### Positive selection detection

To examine whether *ApoD* duplicates underwent adaptive sequence evolution, a branch-site model was used to test positive selection affecting a few sites along the target lineages (called foreground branches) in codeml within PAML [110, 111]. The rates of non-synonymous to synonymous substitutions (ω or dN/dS) with a priori partitions for foreground branches (PAML manual) were calculated. Considering the very dynamic evolutionary pattern of *ApoD* genes, we tested for positive selection in every branch in each species. In this case, we designated each branch as the foreground branch to run the branch-site model multiple times. Noticeably, there are two different ways to designate the foreground branch in the branch-site model. One way is to designate only the branch as the foreground branch. The other way is to designate the whole clade (all the branches within the clade including the ancestral branch) as the foreground branch. We included both perspectives in our data analyses (10.5061/dryad.39g63v2 [116]). If the whole clade was under positive selection, we labelled all the branches within this clade, including the ancestral branch, with ω > 1 in the results (Fig. 4).

All model comparisons in PAML were performed with fixed branch lengths (fix_blength = 2) derived under the M0 model in PAML. Alignment gaps and ambiguity characters were removed (Cleandata = 1). A likelihood ratio test was used to test for statistical significance. In addition, Bonferroni correction was conducted for multiple-test correction [112]. The BEB was used to identify sites that are under positive selection. Sites under positive selection under BEB with a posterior probability > 0.95 are given in Fig. 5 and 10.5061/dryad.39g63v2 [116].

### Gene expression profile analyses

To determine the expression profiles of *ApoD* duplicates in different fishes, raw available transcriptomic data from spotted gar, zebrafish, cavefish, cod, medaka, tilapia and *A. burtoni* were retrieved from NCBI https://www.ncbi.nlm.nih.gov/ (10.5061/dryad.39g63v2 [116]). Raw reads were mapped to the corresponding cDNAs (http://www.ensembl.org/) to calculate the RPKM (reads per kilobase per million mapped reads) value. qPCR was used to detect the expression profiles of different *ApoD* duplicates in the tissues not included in the available transcriptomes (10.5061/dryad.39g63v2 [116]). Fish samples for qPCR include *A. burtoni* (provided by Prof. Walter Salzburger, University of Basel, Switzerland), stickleback (collected from Nideraach and Romanshorn in Switzerland by Dr. Dario Moser), zebrafish (provided by Prof. Markus Affolter, Biozentrum, University of Basel, Switzerland), tilapia (provided by Prof. Deshou Wang, Southwest University, Chongqing, China) and medaka (provided by Prof. Jing Wei, Southwest University, Chongqing, China). Prior to tissue dissection, specimens were euthanized with MS 222 (Sigma-Aldrich, USA) following an approved procedure (permit nr. 2317 issued by the cantonal veterinary office, Switzerland; Guidelines for the Care and Use of Laboratory Animals prescribed by the Regulation of Animal Experimentation of Chongqing, China). RNA isolation was performed according to the TRIzol protocol (Invitrogen, USA). DNase treatment was performed with the DNA-free™ Kit (Ambion, Life Technologies, USA). The RNA quantity and quality were determined with a NanoDrop1000 spectrophotometer (Thermo Scientific, USA). cDNA was produced using the High-Capacity RNA-to-cDNA Kit (Applied Biosystems, USA). The housekeeping gene elongation factor 1 alpha *(elfa*1*)* [113], ubiquitin (*ubc*) [114] and ribosomal protein L7 (*rpl7*) [115] were used as endogenous controls. qPCR was performed on a StepOnePlus™ Real-Time PCR system (Applied Biosystems, Life Technologies) using the SYBR Green Master Mix (Roche, Switzerland) with an annealing temperature of 58 °C and following the manufacturer’s protocols. Primers are available in 10.5061/dryad.39g63v2 [116].

## Abbreviations

ApoD: Apolipoprotein D
BEB: Bayes empirical Bayes
chr: Chromosome
elfa1: Elongation factor 1 alpha
LG: Linkage group
ML: Maximum likelihood
ORF: Open reading frame
qPCR: Quantitative polymerase chain reaction
RPKM: Reads per kilobase per million mapped reads
rpl7: Ribosomal protein L7
SMART: Simple Modular Architecture Research Tool
TGD: Teleost genome duplication
ubc: Ubiquitin
WGD: Whole genome duplication
